# Allergen‐specific immunotherapy by recombinant Der P1 allergen‐derived peptide‐based vaccine in an allergic mouse model

**DOI:** 10.1002/iid3.878

**Published:** 2023-06-09

**Authors:** Soheila Asoudeh Moghanloo, Mohsen Forouzanfar, Mojtaba Jafarinia, Mohammad R. Fazlollahi, Gholam Ali Kardar

**Affiliations:** ^1^ Department of Molecular Genetics, Marvdasht Branch Islamic Azad University Marvdasht Iran; ^2^ Immunology, Asthma and Allergy Research Institute (IAARI) Tehran University of Medical Sciences Tehran Iran; ^3^ Department of Medical Biotechnology, School of Advanced Technologies in Medicine Tehran University of Medical Sciences Tehran Iran

**Keywords:** allergen immunotherapy, house dust mite, IgE, IgG, IL‐13, IL‐4, peptide based vaccine, rDer p1

## Abstract

**Aim:**

Increased IgE levels have made house dust mite allergens one of the most frequent causes of allergies worldwide. Treatment reduces the IgE antibodies and types two cytokines, namely interleukin‐4 (IL‐4) and IL‐13. Although existing treatments significantly reduce IgE or IL‐4/IL‐13, they are very costly. This study aimed to construct a recombinant protein derived from rDer p1 peptides in the form of an immunotherapy approach and to measure the response of IgE and IgG antibodies.

**Methods:**

The proteins were isolated, purified, and evaluated using the SDS‐PAGE and Bradford test and confirmed by using Western blot. To evaluate immunotherapy efficiency, 24 BALB/C mice were sensitized intraperitoneally with house dust mites (HDM) adsorbed to Aluminum hydroxide (Alum) and randomly divided into four groups of six: control sensitized, HDM extract, rDer p1, and DpTTDp vaccine. To immunization, four groups of random mice were each treated with phosphate‐buffered saline, 100 μg of rDer p1 protein, DpTTDp, or HDM extract, every 3 days. Direct ELISA determined HDM‐specific IgG and IgE subclasses. Data were analyzed in SPSS and Graph pad prism software. Values of *p* < .05 were considered significant.

**Results:**

After immunization of mice, the rDer P1 and recombinant vaccine like HDM extract increased IgG antibody titer and decreased IgE‐dependent reactivity in allergic mice to rDer P1. Also, the levels of inflammatory IL‐4 and IL‐13 cytokines as allergic stimulants decreased.

**Conclusion:**

The use of present available recombinant proteins is considered a viable, cost‐effective, and long‐term option for providing effective HDM allergy immunotherapy vaccines without side effects.

## Graphical abstract

House dust mite (HDM) allergens are among the most common causes of allergies worldwide due to increased IgE. Immunotherapy treatment reduces the IgE antibodies and types two cytokines, namely interleukin‐4 (IL‐4) and IL‐13. The use of present available recombinant proteins is considered a viable, cost‐effective, and long‐term option for providing effective HDM allergy immunotherapy vaccines without side effects.

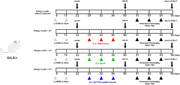

## 1 INTRODUCTION

The prevalence of allergic diseases increases globally because of their severity and complexity, which has launched a global health crisis.^1^ Allergic airway inflammation is a type of immune response that affects individuals with allergies, particularly those with asthma, and causes distinct symptoms and physiological changes in the respiratory system. The infiltration of immune cells, including eosinophils, T‐helper type 2 (Th2) cells, mast cells, and basophils, into the airway tissues, is a hallmark of allergic airway inflammation, and these cells release various mediators that contribute to airway inflammation and hyperresponsiveness.^2^ It can cause significant respiratory symptoms and lung function impairment.^3^ More than 50% of allergic patients suffer from an allergic reaction caused by house dust mites (HDMs), which are a primary source of allergens globally.^4^ HDMs can cause severe and chronic symptoms such as asthma, allergic rhinoconjunctivitis, and atopic dermatitis.^5^, ^6^ HDM has several major allergens, one of which is group 1 *Dermatophagoides pteronyssinus* (Der p1) protein, which is one of the most vital proteins that are responsible for more than 90% of allergies and sensitivities in patients with HDM.^7^ Der p 1, which demonstrates IgE antibody‐binding frequencies of over 80%, was the initial allergen to be identified in HDMs.^8^

Also, Der p2 and Der p23 are two allergenic proteins produced by the HDMs Der p1. Der p1 has differences with Der p2 and Der p23. These differences generally include: Der p1 is a larger protein than Der p2 and Der p23 proteins and therefore has a greater allergenicity effect. Der p1 is considered the most potent allergen among the three proteins and is responsible for inducing allergic reactions in the majority of individuals sensitized to HDMs. Der p2 and Der p23 are also allergenic but to a lesser extent compared to Der p1.^9^ Der p1 is a cysteine protease that can digest proteins in the human airway, leading to inflammation and allergic symptoms. Der p2 and Der p23 are not proteases but are believed to play a role in binding to and activating the immune system cells responsible for triggering allergic reactions. Der p1 is a highly conserved protein with little genetic variation among different strains of Der p1. In contrast, Der p2 and Der p23 exhibit greater genetic diversity, with different variants of these proteins found in different strains of the mite.^9^, ^10^ The only treatment that induces long‐term responses against IgE allergies is allergen‐specific immunotherapy (AIT).^11^, ^12^, ^13^ There have been clinical studies performed recently to improve the mechanisms and approaches of AIT regarding HDM as one of the essential allergen sources using recombinant methods and peptide vaccines.^14^ Several studies have shown that epitope‐based peptides of major allergens are suitable to effectively treat allergies.^15^, ^16^, ^17^, ^18^, ^19^, ^20^ These synthetic peptide‐based vaccines offer a promising alternative to traditional allergy immunotherapy methods and may provide a safer and more efficient approach to treating HDM allergies.

In this study, we used a B‐cell epitope vaccine named DpTTDp for HDM immunotherapy, with the aim of decreasing IgE and T‐cell reactivity. The vaccine includes the Der p 1 sequence, which is a hypoallergenic area with some B‐cell epitopes and lacking T‐cell epitopes. Two copies of this hypoallergenic peptide were combined with a partial fragment C of tetanus toxoid (TT) to make them immunogenic and help T‐cells. Also, this vaccine contains a partial C fragment of TT molecule as a carrier because it is reported to be nontoxic and immunogenic and can be expressed in prokaryotic systems with high efficiency.^21^ Then, experimental techniques were used to compare and evaluate the fusion vaccine against recombinant Der p 1 (rDer p 1) and HDM allergen extract.

## 2 MATERIALS AND METHODS

### 2.1 Construction of the recombinant fusion vaccine

The recombinant fusion vaccine was developed by our previous group.^22^ The final sequence of the vaccine is DpTTDp. To construct the recombinant fusion vaccine, two predicted hypoallergenic peptides were fused together with a partial fragment C of the TT sequence, which is composed of 155 amino acids. Short linker sequences (GGGGS) were added to the N‐ and C‐terminus of the TT sequence. Additionally, a Hexa‐histidine tag was attached to the C‐terminus of the final protein sequence to simplify its purification process.^22^

### 2.2 Expression and purification of DpTTDp vaccine and rDer P1 proteins

To the expression of rDer P1 and DpTTDp according to our previous work,^22^ a positive BL21 clone was selected and inoculated into 150 mL of LB medium with kanamycin antibiotic and cultured overnight. At an OD of 0.6 in 600 nm, the culture was induced with 1 mM isopropyl‐beta‐thiogalactopyranoside (IPTG). It was incubated and shacked 200 rpm at 28°C for 18 h. The resulting bacterial cells were lysed in a 5 mL lysis buffer and centrifuged at 4°C at 12,000 rpm for 30 min. The pellet dissolved with denaturant buffer (100 mM NaH_2_PO_4_ + 10 mM Tris‐HCL + 8 M Urea; Adjust pH to 8.0 using NaOH). After dissolution, to increase its purity, the sample was centrifuged again at 14,000 rpm for 10 min, then Affinity chromatography was performed by the resulting supernatant. Dialysis was performed to refold the proteins from urea, with descending concentrations of 8, 4, 2, and 1 M, respectively, overnight.

### 2.3 Affinity chromatography

Ni‐IDA column enables fast and easy purification of recombinant proteins labeled with polyhistidine. This operation is performed by metal ion affinity chromatography. The final equilibration of the DpTTDp proteins was loaded with 2 mL of Ni‐IDA (Ni‐IDA) (ARG Biotech, Iran). Finally, a washing step was performed with dissolution on buffer containing 25 mM imidazole to remove impurities and nonspecific proteins. The same steps were performed for rDer p1 protein and labeled 6‐histidine at the C end of it separately expressed and purified in *E. coli* BL21 (DE3) cells.

### 2.4 SDS‐PAGE electrophoresis

SDS‐PAGE is a commonly used technique for analyzing protein mixtures, enabling qualitative analysis, and purification of proteins. Since the separation of proteins is based on their size, this method can also be used to determine the relative molecular mass of proteins. Polyacrylamide gel electrophoresis was performed to evaluate the purification quality of the DpTTDp proteins and rDer p1 proteins. Thus, 40 µL of the purified samples were mixed with 10 µL loading buffer containing 2‐mercaptoethanol, and after boiling at 12%, SDS‐PAGE gel was loaded and finally was stained by Coomassie blue. Finally, another buffer‐washing step was performed, and the verification of recombinant proteins was confirmed through 12% SDS‐PAGE.

### 2.5 HDM extract

SDS PAGE and Coomassie blue staining were used for HDM extract purchased from Greer Laboratory to determine the content and quality of extract proteins under reducing conditions. All dilutions were prepared using phosphate‐buffered saline (PBS), and the extract was stored at 4°C.

### 2.6 Animals

Twenty‐four female BALB/C mice, 6 weeks of age, were purchased from Iran Pasteur Institute and provided with food and water. The project was conducted in compliance with ethical principles and national standards for medical research in Iran. The Institutional Research Ethics Committee in Iran granted approval for the study. (IR.IAU.M.REC.1399.43‐2020).

### 2.7 Sensitization and allergic airway inflammation model and vaccination protocol

**Figure 1:**
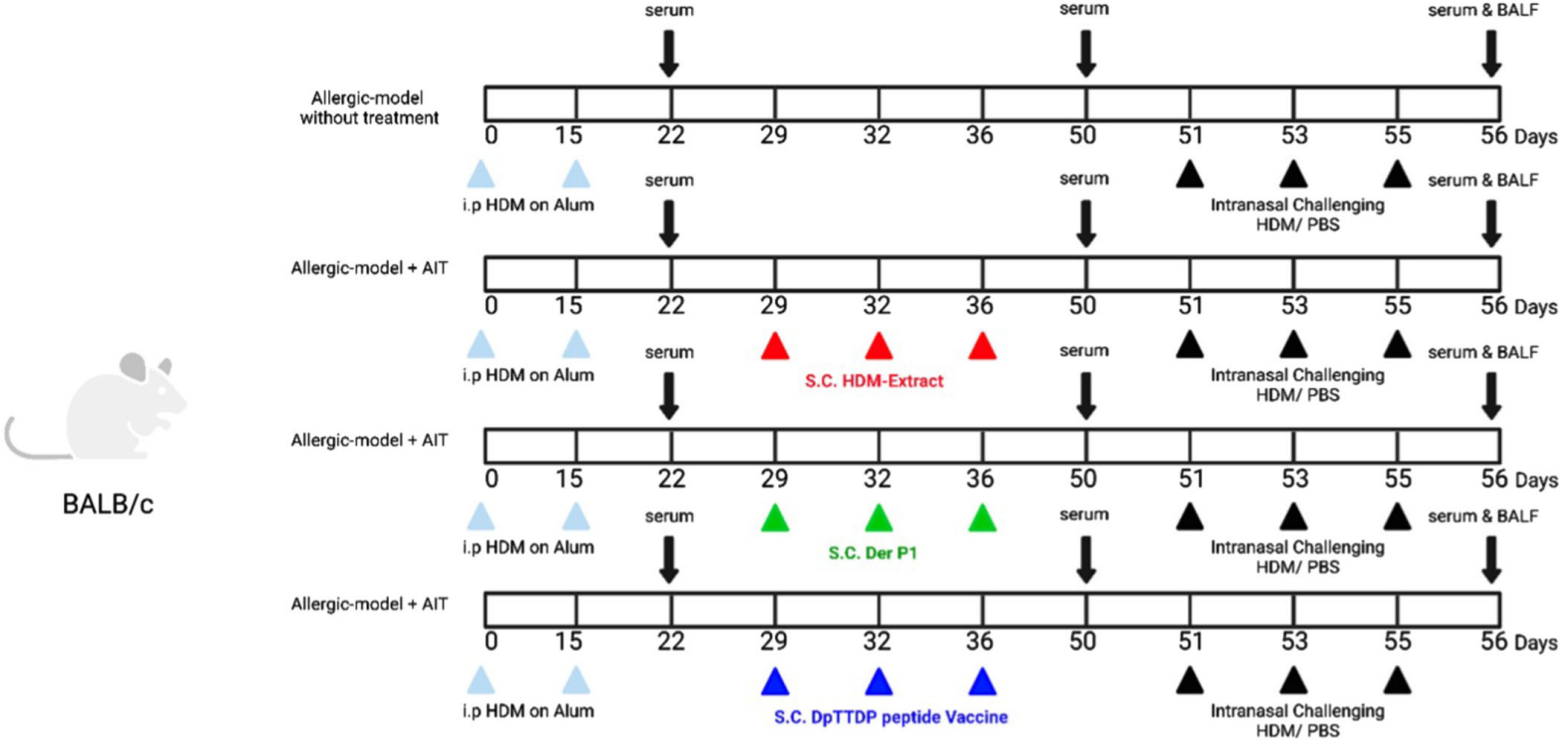
Experimental scheme of AIT model. BALF fluid and serum from Allergic‐model without treatment, Allergic model+ AIT (HDM‐Extract), Allergic model+ AIT (rDerP1), and Allergic model+ AIT (DpTTDp peptide Vaccine) mice were analyzed (*n* = 6 per group). First, to sensitize HDM in mice, on Days 0 and 15, 10 μg of HDM with 100 μL of alum was injected intraperitoneally into each mouse. SCIT immunotherapy was performed on Days 29, 32, and 36 using the S. C. injection method. In the next stage, an allergy challenge was performed on Days 51, 53, and 55. For this purpose, each group of mice inhaled 15 mL of HDM + PBS with a nebulizer every day for 20 min. Then, blood samples were taken in the 56th and after the allergy challenge stage. Blood samples were also taken on Days 22, 50, and 56 to measure antibody levels. After the last blood draws on Day 56, the mice were anesthetized with ether, and BALF was collected to measure interleukin levels 4 and 13 (SPSS v.20; GraphPad Prism version 8.4.3). BALF, bronchoalveolar lavage fluid; HDM, house dust mites; PBS, phosphate‐buffered saline.

The mice that were purchased were randomly assigned to four groups of six; including (i) control, (ii) HDM extract, (iii) rDer p1, and (v) DpTTDp peptide vaccine. The mice used in the study were housed in a controlled environment that was specifically pathogen‐free and had a temperature control system in place. The mice were kept on a strict 12 h light‐dark cycle and were provided food and water as per the animal care guidelines of TUMS. The term “allergic model” was used in the manuscript to refer to the HDM‐Alum‐sensitized allergic airway inflammation mouse model. The mice were sensitized by intraperitoneal injection with a mixture of 10 μg HDM adsorbed to 1 mg/mL alum adjuvant in 100 μL as the total volume on Days 0 and 15 to induce allergic airway inflammation. Continued mice were done treatment according to the following protocol (Figure 1).

Group 1 (positive control *n* = 6): Allergic model. This group received normal saline.

Group 2 (HDM‐Extract *n* = 6): Allergic models were immunized with 100 μg HDM‐Extract through the subcutaneous injection on Days 29, 32, and 36.

Group 3 (rDerP1 *n* = 6): Allergic models were immunized with 100 μg rDerP1 through the subcutaneous injection on Days 29, 32, and 36.

Group 4 (DpTTDp peptide vaccine *n* = 6): Allergic models were immunized with 100 μg DpTTDp peptide vaccine through the subcutaneous injection on Days 29, 32, and 36.

### 2.8 Allergen challenge

All 4 groups of mice were challenged by HDM respiration. In the challenging step, an ultrasonic nebulizer (NE‐C900; Omron Corp.) was used to administer aerosols at a constant pressure inside a 3500 cm^3^ chamber on Days 51, 53, and 55. On Day 56, the mice were killed and their blood and bronchoalveolar lavage fluid (BALF), were collected for subsequent analysis.

### 2.9 BALF preparation

On Day 56, six mice from each group were anesthetized using ether, and blood samples were collected to obtain serum. To prepare BALF, the mice were killed, and their tracheas were cannulated to collect the BALF‐containing cells. Both blood and BALF samples were collected for subsequent analysis. The specific IgE and IgG antibodies were measured in the serum samples, while the inflammatory cytokines interleukin (IL‐4) and IL‐13 were measured in the BALF samples.

### 2.10 Specific IgE & IgG measurement from sera by ELISA

ELISA was performed for immunological studies, including measuring specific IgE and IgG antibody levels against recombinant Derp1. A solution of recombinant DerP1 at a concentration of 1 μg/mL was prepared by reconstituting it in PBS. Then, 100 μL of this solution was added to every well in a Nunc MaxiSorp flat‐bottom 96‐well plate (Thermo Fisher Scientific). After sealing the plate, it was refrigerated at 4°C overnight. The following day, the plate was washed with PBS containing 0.1% (v/v) Tween 20 (Wako Junyaku; further designated as PBS‐T), and then the blocking buffer, which was composed of PBS‐T containing 3% (w/v) skim milk, was added to each well. The plate was then incubated at room temperature for 1 h. Following the PBS‐T washing step, 100 μL of diluted mice sera (at 1/25, 1/50, 1/100, and 1/250 using PBS‐T) was added to each well and incubated for 1.5 h at 37°C. Following that, a PBS‐T‐containing solution was used for washing the plate and adding Gout antiMouse IgE b and Gout anti‐Mouse IgG (Abcam) as primary antibodies. After 1.5 h at 37°C, the plate was again washed with PBS‐T. Then, a 100 μL of Rabbit antiGout IgG‐HRP conjugate as a secondary antibody (Abcam) at 1/1000 dilution in PBS‐T was added to each well. Following 1 h of incubation at 37°C, the plate was washed with PBS‐T again. In the development step, 100 μL of 1‐Step Ultra TMB‐ELISA (Thermo Fisher Scientific), was added and incubated at room temperature for 15 min in the dark. Fifty microliters of 1 N H_2_SO_4_ solution was added to terminate the reaction. A Stat Fax 2100 Microplate Reader was used to measure the absorbance at 450 nm in comparison to the 630 nm reference wavelength.

### 2.11 Measurement of IL‐4 and IL‐13 from BALF by ELISA

The concentrations of inflammatory IL‐4 and IL‐13 cytokines in BALF were quantified using commercial ELISA kits based on the manufacturer's protocols (eBioscience).

### 2.12 Inflammatory cell counting by hemocytometer

To determine the proportion of inflammatory cells in BALF, H&E staining was performed. Around 200 cells were counted to determine the percentage of eosinophils, macrophages, neutrophils, and lymphocytes. The total number of eosinophils, macrophages, neutrophils, and lymphocytes in each milliliter of BAL fluid was calculated by total cell counts and percentage values.

### 2.13 Statistical analysis

The statistical analysis of the data was performed using the SPSS v.20 software package (IBM Corp.). The Mann−Whitney *U* test was used to determine differences in the levels of antibodies and cytokines induced by immunization of mice, and Student's paired *t*‐test was used to compare control and vaccination groups. The results were expressed as mean ± standard deviation, and differences were considered significant when *p* < .05 (∗), *p* < .01 (∗∗), or *p* < .001 (∗∗∗). All experiments were performed in triplicates. Graphs were created using GraphPad Prism software (GraphPad Prism version 8.4.3; GraphPad).

## 3 RESULT

**Figure 2:**
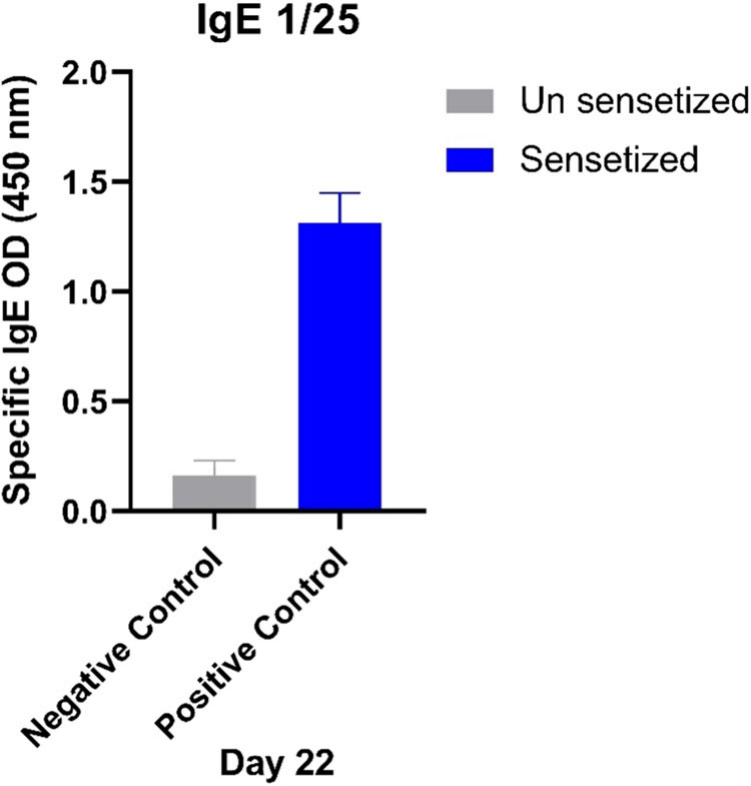
Comparison of IgE level in blood serum of unsensitized mice and sensitized mice: The negative control mice group were not sensitized. But the positive control group received the HDM allergen in the form of intraperitoneal injection and were sensitized. The level of IgE is low in the negative control group and increased in all sensitive groups (*p* < .001) (SPSS v.20; GraphPad Prism version 8.4.3). HDM, house dust mites.

The level of IgE in the mice of the first group that were sensitized was compared with the non‐sensitized mice that were the negative control group. As a result, after performing the ELISA test and checking the IgE level in both groups of mice, it was observed that the level of IgE was low in the group of negative control mice and the level of IgE was increased significantly in all sensitized groups (*p* < .001), and this indicates that is the allergic models were prepared correctly (Figure 2).

The IgE levels in the blood serum of the allergic mouse model showed a statistically significant difference between the control group and the other groups (*p < .05, **p < .01, ***p < .005). The reduction of IgE was clearly observed in the group of mice vaccinated with the DpTTDp vaccine compared to non‐vaccinated mice after SCIT immunotherapy. Therefore, it can be concluded that increasing the dose of the DpTTDp vaccine can completely inhibit the binding of mouse serum IgE to the rDer p1 allergen (Figure 4A,B). The results showed an increase in IgG in the group of mice vaccinated with the DpTTDp vaccine compared to non‐vaccinated mice after SCIT immunotherapy. Also, HDM extract induces the least specific antibodies while rDer p1 induces a higher level of IgG compared to the DpTTDp vaccine. Therefore, immunotherapy with each of the three HDM‐Extract, rDer P1, and DpTTDp peptide vaccine‐induced highly significant increases of IgG antibodies specific for the major HDMs allergens (Figure 4C,D). The IgG levels in the blood serum of the allergic mouse model showed a statistically significant difference in the control groups and the other groups (*p < .05, **p < .01, ***p < .005 (Table 1).

**Table 1:** The comparison of allergen format efficiency.

IgG 1/250 Days 50	HDM	rDerP1	Vaccine
HDM		0.015	0.932
rDerP1	0.015		0.078
Vaccine	0.932	0.078	

IgE 1/50 Days 50	HDM	rDerP1	Vaccine
HDM		0.909	0.001
rDerP1	0.909		0.006
Vaccine	0.001	0.006	

IgG 1/100 Days 56	HDM	rDerP1	Vaccine
HDM		0.469	0.613
rDerP1	0.469		0.046
Vaccine	0.613	0.046	

IgE 1/50 Days 56	HDM	rDerP1	Vaccine
HDM		0.9	0.204
rDerP1	0.9		0.096
Vaccine	0.204	0.096	

Abbreviation: HDM, house dust mites.

Following the HDM challenge the amount of IL‐4 and IL‐13 cytokines present in the BAL fluid was assessed. Recombinants and peptide vaccines significantly reduced IL‐4 (Figure 4A) and IL‐13 in lung lavage fluid (Figure 4B). Additionally, the concentrations of IL‐4 and IL‐13 in the BALF significantly increased in control allergic mice without AIT treatment compared to Allergy‐AIT‐extract treatment, while HDM‐Extract‐AIT treatment significantly reduced the levels of IL‐4 and IL‐13. Similarly, BALF levels of IL‐4 were significantly lower in the Allergy‐AIT‐rDer p1 and Allergy‐AIT‐DpTTDp vaccine compared to control allergic mice without AIT treatment, and there was a tendency toward a decrease in the concentrations of IL‐4 (Figure 4A) and IL‐13 (Figure 4B). AIT resulted in a significant reduction in the secretion of Th2‐cytokines IL‐4 and IL‐13 into the BAL fluid. Additionally, there was a tendency towards a further decrease in their concentrations following treatment with the rDer P1 and DpTTDp vaccine.

By counting Inflammatory cells, was observed a significant increase in cell infiltration, particularly eosinophils, in the BAL fluid of mice sensitized and challenged with HDM (Figures 1 and 5). The treatment with AIT‐rDerp1 and AIT‐DpTTDp vaccine resulted in significant further suppression of the BAL infiltrate (Figure 5). Furthermore, AIT improved HDM‐induced inhibition of eosinophil BAL cell infiltration compared to allergic mice. Administration of rDerp1 and DpTTDp vaccine resulted in an additional significant reduction of eosinophil infiltration. The reduction in eosinophil infiltration was accompanied by a significant relative increase in macrophages, neutrophils, and lymphocytes.

## 4 DISCUSSION

Allergic airway inflammation is an immune response that affects the respiratory system and is commonly seen in individuals with allergies, particularly asthma. HDM allergens, which are IgE allergy triggers, affect 50% of allergic people worldwide.^17^ Apart from being managed using pharmacotherapy, HDM allergy has been treated using allergen extracts from the mites.^23^, ^24^ The advent of recombinant DNA technology has seen the targeting of allergenic regions of allergens for immunotherapy of aero‐allergies.^25^, ^26^ The therapeutics design and in vivo & in vitro application of recombinant hypoallergens have been strongly integrated with immunoinformatics.^27^, ^28^ The B‐cell epitope vaccine DpTTDp for HDM allergy used in this study was designed using bioinformatics.^22^ The vaccine consists of a 34‐amino acid (hypoallergenic) sequence derived from Der p1 (lacking the specific T cell epitope Der p1), a major HDM allergen responsible for 90% of sensitivities in HDM‐allergic subjects, and two copies of a hypoallergenic peptide conjugated to a partial TT carrier molecule. Finally, GGGGS linkers were used to separate hypoallergenic segments with the TT carrier. The choice of TT structure as the carrier molecule in this vaccine is due to its non‐toxicity, immunogenicity, high performance, and also expression in *E. coli* systems.^21^ For this study, the vaccine was expressed in *E. coli* BL21 cells and then purified for further evaluation.

**Figure 3:**
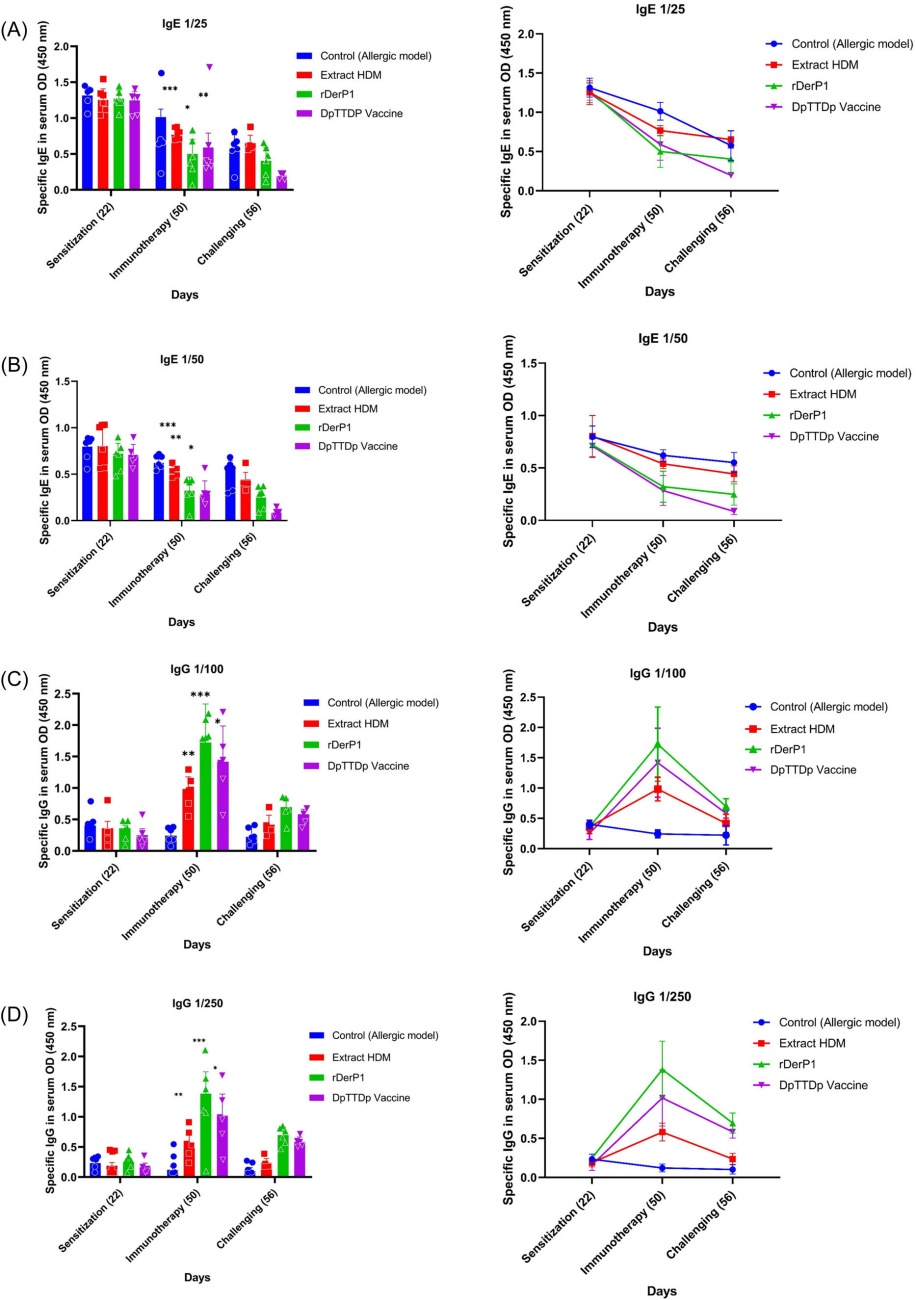
Comparison of specific IgE and IgG titers in mice sera obtained by immunization with rDer p1, house dust mite and DpTTDp vaccine. Mice in groups of four were injected subcutaneously with DpTTDp vaccine, HDM extract, and rDer p1 proteins at a dose of 100 μg/mouse. Sera samples before immunization on Day 22 (preimmunization) and after immunization on Day 50 and after challenging (intranasal inhalation) on Day 56 were tested for IgE and IgG responses using ELISA after coating wells with rDer p1. The mean levels of allergen specific IgE and IgG optical densities (recorded at optical density 450 nm) are shown on the *y*‐axis (IgE sera dilutions 1:25 and 1:50 and IgG sera dilutions 1:100 and 1:250). (A) The peptide vaccine led to a significant decrease in the relative level of 1/25 IgE in mice sera. (B) The peptide vaccine led to a significant decrease in the relative level of 1/50 IgE in mice sera. (C) The peptide vaccine led to a significant increase in the relative level of 1/100 IgG in mice sera. (D) The peptide vaccine led to a significant increase in the relative level of 1/250 IgG in mice sera (Table 1). *p < .05, **p < .01, ***p < .005 (SPSS v.20; GraphPad Prism version 8.4.3). HDM, house dust mites.

**Figure 4:**
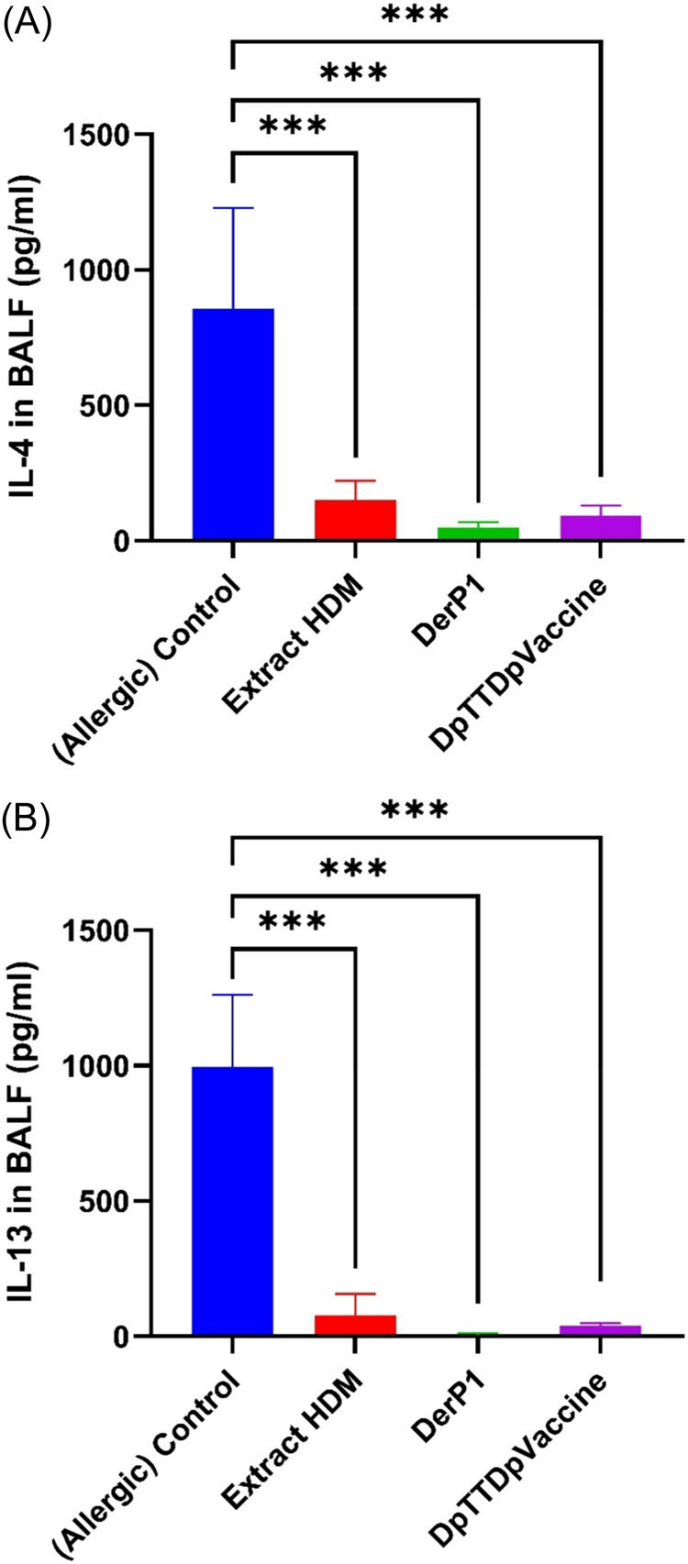
Results of IL‐4 and IL‐13 levels in the BALF of allergic mice in studied groups. Recombinants and peptide vaccines significantly reduced interleukin levels 4 (A) and 13 in lung lavage fluid (B). ***p < .005 (SPSS v.20; GraphPad Prism version 8.4.3). BALF, bronchoalveolar lavage fluid; IL, interleukin.

**Figure 5:**
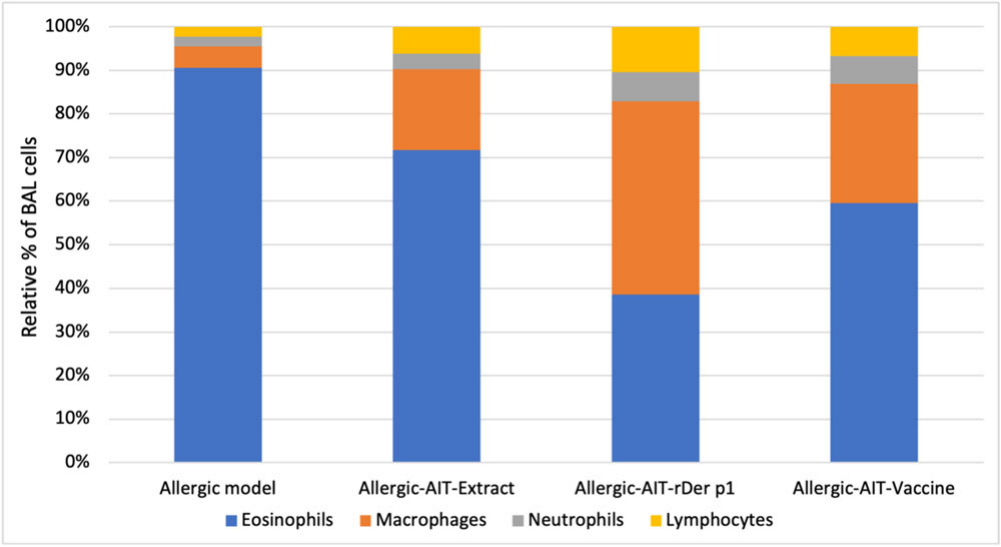
Analysis of the cellular BAL fluid of mice (Allergic model, Allergic‐AIT‐Extract, Allergic‐AIT‐rDer P1, and Allergic‐AIT‐DpTTDp vaccine) after HDM‐aerosol challenge. Percentages of eosinophils, macrophages, neutrophils, and lymphocytes within the total BAL cells. Cells were fixed and stained with H&E. Around 200 cells were counted and analyzed with a hemocytometer under a microscope. Values are presented as mean ± SD; *n* = 6 per group (Microsoft Excel 2019). HDM, house dust mites.

In this study, we compared rDer p 1, HDM extract, and DpTTDp vaccine according to their ability to induce T cell proliferation in the serum of HDM‐allergic mice, as well as the level of cytokines IL‐4 and IL‐13. We also investigated the penetration of inflammatory cells such as eosinophils in the BAL fluid of mice. Our strategy was to investigate the clinical efficacy of AIT using the induction of an IgG antibody response, which would subsequently inhibit the binding of allergens to specific IgE antibodies. The clinical efficacy of AIT is related to the level of IgG, while symptoms in allergic diseases are inversely related to the ratio of IgG to IgE, indicating the relevance of the IgG response induced during AIT. Therefore, we calculated the changes in IgG level and IgE level and compared them between groups (Figure 3). We compared these IgG/IgE ratios both before and after immunotherapy and after challenging with the positive control. Our vaccine was effective in eliminating IgE‐reactivity, as demonstrated by our findings (Figure 3A,B). Through the immunization of mice with the DpTTDp vaccine, we were able to induce blocking IgGs, which suggests that the TT carrier possesses immunomodulatory capabilities (Figure 3C,D). Compared to the DpTTDp vaccine, the HDM extract induced the lowest specific antibodies, while rDer p1 induced higher levels of IgGs (Figure 3C,D). This disparity might be due to the fact that the 34 amino acid hypoallergenic peptides in the DpTTDp vaccine did not encompass the full epitope spectra of the Der p1 sequence, unlike the complete and purely expressed rDer p1 protein. Consequently, the DpTTDp vaccine may have generated IgGs that covered fewer Der P1 epitopes. Based on these results, the level of IgG has increased after immunotherapy (Figure 3C,D). The induction of a strong IgG response in this study is also in line with the findings of previous studies.^29^, ^30^, ^31^ An important point in the obtained results is the reduction of the specific IgG titer after the challenge. This is probably due to the low number of immunizations, and if we increase the number of doses and the time to more than 2 weeks, this reduction will not be seen. Increasing the immunization dose can easily resolve the minor problem of low immunizations. Due to the hypoallergenic nature of the DpTTDp vaccine and its inability to activate T‐cells effectively, IgG titer can be safely increased without worrying about any IgE‐dependent or IgE‐independent side effects. The study results indicate that the peptide vaccine was successful in stimulating an IgG response while simultaneously suppressing the expression of the IL‐4 and IL‐13 cytokines that typically stimulate IgE (Figure 4A,B). It was also more effective in reducing IL‐4 (Figure 4A) AND IL‐13 (Figure 4B) cytokines than HDM extract, which was in line with the Fanuel et al. study.^22^ On the other hand, a decrease in the level of cytokines IL‐4 and IL‐13 in BALF was observed in similar studies using DerP2 peptides by Wang, as well as studies by Xiao‐Dong et al. separately. They have also introduced this peptide as a candidate for allergy vaccines.^18^, ^19^ In our previous study, the results of ELISA inhibition experiments indicated that the DpTTDp vaccine had a considerable capability to inhibit the binding of IgE antibodies to allergens in sera obtained from patients with an HDM allergy. Increasing the dose of the DpTTDp vaccine can lead to the inhibition of IgE binding of patients' serum to rDer p1 allergen (35). However, the fact that DpTTDp was able to reduce IgE reactivity even in mice serum means it has some meaningful advantage. Also, according to the obtained results, this vaccine can effectively reduce the allergic inflammation of the airways and lungs in mice after immunotherapy. The sensitization and challenge with HDM caused a substantial cell infiltrate in the BAL fluid, with a dominant presence of eosinophils (Figure 5). However, treatment with AIT‐rDerp1 and AIT‐DpTTDp vaccine resulted in significant suppression of the BAL infiltrate. Additionally, AIT was found to improve the HDM‐induced inhibition of eosinophil BAL cell infiltration compared to allergic mice. The administration of rDerp1 and DpTTDp vaccine resulted in a further significant reduction of eosinophil infiltration, accompanied by a significant relative increase of macrophages, neutrophils, and lymphocytes (Figure 5). Examination of BAL fluid of mice after the HDM‐aerosol challenge phase showed that eosinophil infiltration was significantly reduced in the DpTTDp vaccine and rDerP1 treatment compared to the HDM allergen. This decrease in eosinophil infiltration is associated with the inhibition of airway inflammation. This review shows the ability of the vaccine to prevent HDM allergic asthma by inhibiting airway inflammation. which is in accordance with the findings of Xiao‐Dong et al. (Figure 5).^19^, ^32^

Therefore, in accordance with the results of Fanuel et al. study, HDM extract induced the lowest specific antibodies to peptide vaccine and Der p1, indicating the superiority of peptide vaccines over HDM extract. Hence, hypoallergens might be potentially useful for prophylactic purposes as they cannot induce allergen‐specific T‐cell responses. In addition, they can be combined with carrier molecules to offer protection against diseases caused by pathogens like hepatitis, rhinoviruses, and cholera.^30^, ^31^, ^33^ These carrier molecules can carry hypoallergenic peptides and provide immunity against the pathogen's proteins.

## 5 CONCLUSION

Overall, the use of recombinant peptide vaccines for allergen immunotherapy shows promise as a safe and effective alternative to traditional natural allergen extract vaccines. In conclusion, the DpTTDp peptide vaccine was shown to induce a strong IgG response while inhibiting the expression of IL‐4 and IL‐13 cytokines as IgE stimulants, effectively reducing airway inflammation in mice, which are according to the research results of Valenta et al.^34^ This protein represents a promising vaccine for HDM allergy immunotherapy. The use of recombinant B‐cell epitope vaccines offers several advantages over traditional allergen extract vaccines and holds great potential for future development and optimization. Despite this, the vaccine development strategy presented in this study could potentially be extended to other allergens and species for allergen immunotherapy.

## 6 FUTURE PERSPECTIVE

Future studies could explore the possibility of combining recombinant B‐cell epitope vaccines with T‐cell epitope vaccines to enhance the therapeutic effect and generate a more comprehensive immune response. Since, this study made use of only one major HDM allergen Der p1, therefore in future studies, there is a need to include hypoallergenic peptides from other relevant major allergens such as Der p2 and Der p23 when designing recombinant B‐cell epitope vaccines to cover as much spectrum of most clinically relevant HDM allergens as possible^35^ that may bring in a lot of opportunities to improve diagnosis and treatment of allergies.

## AUTHOR CONTRIBUTIONS

Gholam Ali Kardar conceived the conceptualization and was responsible for the design and development of the methodology. Gholam Ali Kardar and Mohammad R. Fazlollahi provided critical materials. Soheila Asoudeh Moghanloo performed the experiments. Soheila Asoudeh Moghanloo, Mohsen Forouzanfar, and Mojtaba Jafarinia analyzed the data. Soheila Asoudeh Moghanloo and Gholam Ali Kardar wrote and edited the manuscript. All authors have read and agreed to the published version of the manuscript.

## CONFLICT OF INTEREST STATEMENT

The authors declare no conflict of interest.

## ETHICS STATEMENT

The project was found to be in accordance with the ethical principles and the national norms and standards for conducting Medical Research in Iran. The study was approved by the Institutional Research Ethics Committee in Iran. (IR.IAU.M.REC.1399.43‐2020). The experiments were performed according to the ARRIVE guidelines.

## Data Availability

The data sets used or analyzed during the current study are available from the corresponding author on reasonable request.
